# Single-port Robotic Prostatectomy with Neuraxial Anesthesia and Virtual Reality Support: Combining Technologies To Minimize Surgical Impact

**DOI:** 10.1016/j.euros.2025.11.003

**Published:** 2025-11-24

**Authors:** Daniele Amparore, Gabriele Bignante, Enrico Checcucci, Alessandra Saliva, Paolo Alessio, Gabriele Volpi, Michele Sica, Saverio Liguori, Michele Ortenzi, Stefano Zuccolin, Stefano Alba, Alessandro Cerutti, Francesco Porpiglia

**Affiliations:** a Department of Oncology, University of Turin, Orbassano, Italy; b Division of Urology, Department of Surgery, Candiolo Cancer Institute FPO-IRCCS, Candiolo, Italy; c Division of Anesthesiology, Department of Surgery, Candiolo Cancer Institute FPO-IRCCS, Candiolo, Italy; d Department of Urology, Romolo Hospital, Rocca di Neto, Italy

**Keywords:** Single-port surgery, Extraperitoneal access, Spinal anesthesia, Virtual reality, Anxiety reduction, Enhanced recovery

## Abstract

Minimally invasive surgery continues to evolve to reduce the surgical and anesthetic burden for patients. We present the first prospective pilot case series to assess the feasibility and safety of combining extraperitoneal single-port robot-assisted radical prostatectomy (SP-RARP) with neuraxial anesthesia and intraoperative virtual reality (VR) support. Ten consecutive patients with low-risk prostate cancer underwent SP-RARP under combined spinal-epidural anesthesia and immersive VR distraction offered by the HypnoVR device to enhance patient comfort and reduce anxiety during surgery. Dedicated questionnaires focused on intraoperative HypnoVR tolerability and the patient experience. Data for perioperative parameters and anxiety trends were collected. All procedures were successfully completed without conversion to general anesthesia. Hemodynamic stability was maintained, with only two transient hypotensive episodes managed pharmacologically. Median operative time was 90 min and median hospital stay was 2 d, with no intraoperative or postoperative complications. Pain scores remained negligible (Visual Analog Scale 0/10) and no involuntary movements were reported. Nine patients (90%) completed surgery wearing the HypnoVR visor; most reported better comfort and lower anxiety. State-Trait Anxiety Inventory scores significantly decreased from before surgery to the 24-h postoperative assessment, and Health-Information Technology Usability Evaluation Scale scores confirmed high usability. The study demonstrates that SP-RARP under neuraxial anesthesia combined with VR is safe, feasible, and well accepted, and supports further investigation to validate the impact of this approach on recovery and patient-centered outcomes.

## 1 Case series

Minimally invasive surgery has transformed the management of localized prostate cancer (PC) and offers shorter recovery times, lower morbidity, and better perioperative outcomes in comparison to open techniques [1]. The introduction of single-port (SP) robotic platforms has advanced this evolution by allowing an extraperitoneal approach with smaller incisions, minimal insufflation, and better cosmetic outcomes. Crucially, SP surgery avoids the peritoneal cavity and thus eliminates the physiological burden of the pneumoperitoneum and steep Trendelenburg positioning typically needed in conventional intraperitoneal robot-assisted radical prostatectomy (RARP) [2].

This setting represents an opportunity to perform RARP under neuraxial anesthesia, which can potentially reduce the risks associated with general anesthesia such as airway trauma, pulmonary complications, and postoperative nausea or delirium. Neuraxial techniques may also minimize hemodynamic fluctuations and reduce perioperative opioid requirements, in alignment with enhanced recovery principles [3].

In parallel, attention has been directed towards optimizing the patient’s perioperative experience beyond surgical and anesthetic domains. Virtual reality (VR) has emerged as a promising adjunct by providing immersive distraction and anxiety reduction in different surgical settings [4]. For some urological procedures, VR has shown encouraging results in improving comfort during interventions performed under local or regional anesthesia, with potential to reduce the need for additional sedatives [5,6]. However, VR has never been tested in combination with neuraxial anesthesia for major urological procedures such as SP-RARP performed extraperitoneally.

The aim of our study was to test this integrated approach in a cohort of patients with low-risk PC who were candidates for SP-RARP and evaluate its safety, feasibility, and impact on perioperative outcomes and the patient experience.

This single-center, prospective, observational pilot cohort study was conducted between June and September 2025 at Candiolo Cancer Institute. Consecutive patients undergoing SP-RARP under spinal anesthesia combined with VR mask support (HypnoVR mask; HypnoVR, Strasbourg, France) were enrolled. All procedures were performed by a single surgeon (F.P.). Written informed consent was obtained separately for the surgical and anesthetic components. Clinical data were prospectively collected and entered in an institutional review board–approved database (approval number 362/2024).

Eligible patients had low- or intermediate-risk localized PC, prostate volume <80 ml, American Society of Anesthesiologists (ASA) physical status I–III, and body mass index (BMI) <30 kg/m^2^. All cases were discussed at multidisciplinary team meetings and patients were counseled regarding active surveillance, but chose to undergo radical surgery after shared decision-making. Exclusion criteria were previous prostatic surgery, previous surgery in the extraperitoneal pubic space, severe pulmonary disease, obstructive sleep apnea, contraindications to spinal anesthesia (eg, previous spinal surgery, severe discopathy), and claustrophobia or patient refusal to wear a VR mask at the time of the procedure.

The main outcomes were the safety and feasibility of VR mask–assisted neuraxial anesthesia for extraperitoneal SP-RARP, with a focus on intraoperative tolerability and subjective perception of the VR experience. Patient anxiety and pain, as well as anesthesiological and surgical outcomes, were assessed.

**Fig. 1:**
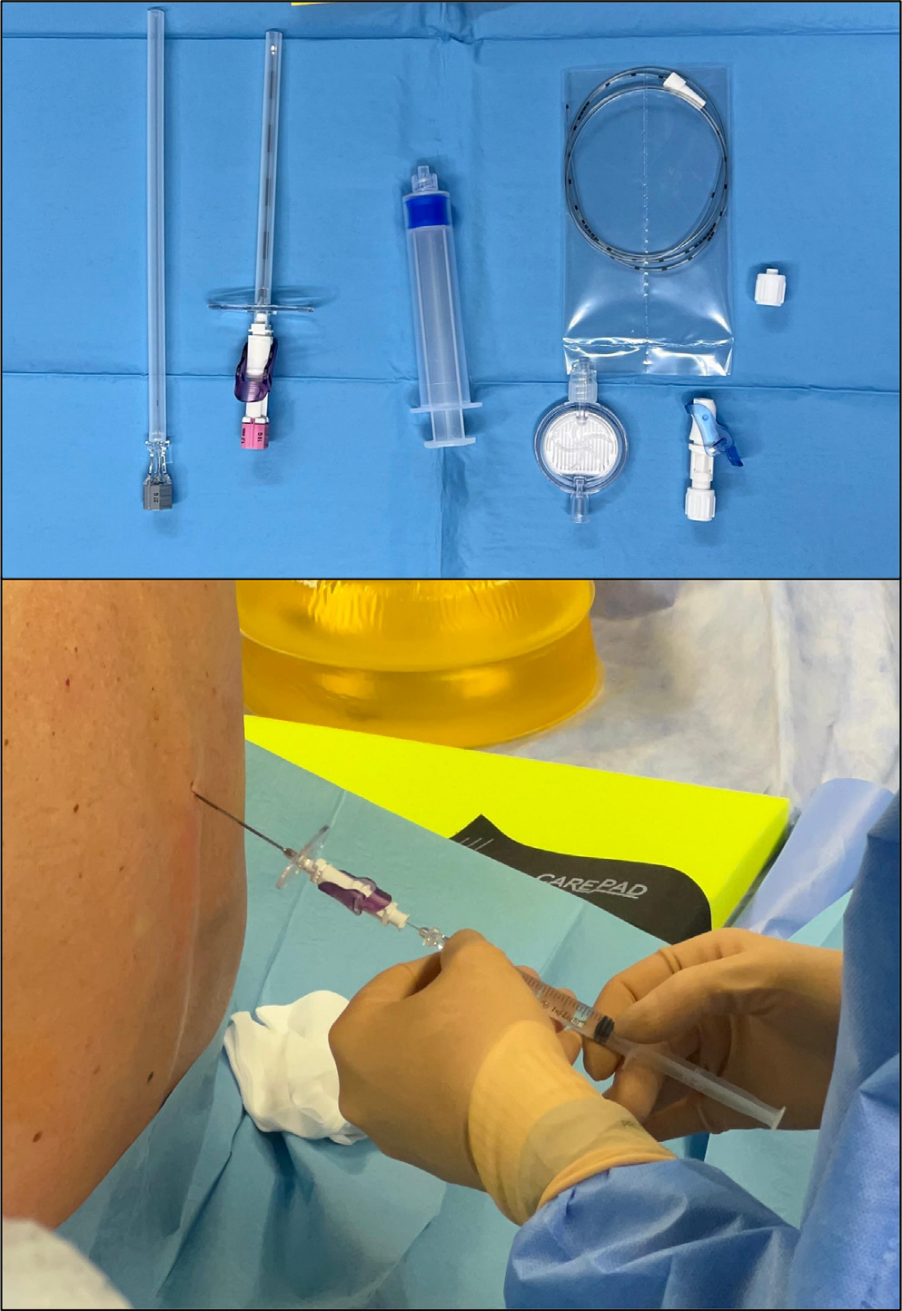
Neuraxial anesthesia kit and administration of combined spinal-epidural anesthesia at the L1–L2 level under sterile conditions.

Combined spinal-epidural anesthesia (CSE) was performed at the L1–L2 or L2–L3 interspace following local infiltration using a spinal injection of 3 ml of levobupivacaine 5 mg/ml and 0.5 ml of fentanyl 50 μg/ml (Fig. 1). The epidural catheter was advanced 6 cm into the epidural space, followed by an epidural injection of 1 ml of levobupivacaine 5 mg/ml. Sedation was maintained with a continuous dexmedetomidine infusion, titrated to achieve a Richmond Agitation-Sedation Scale score of −1. For the surgical procedure, patients were placed in a supine position, breathing spontaneously with supplemental oxygen via a nasal cannula (target SpO_2_ >94%). Continuous end-tidal CO_2_ monitoring was performed using capnography. Fluid maintenance comprised administration of an isotonic crystalloid solution at 3 ml/kg/h. At surgical incision, multimodal intravenous analgesia was initiated with acetaminophen 1 g and parecoxib 40 mg. An elastomeric epidural pump delivering levobupivacaine 0.625 mg/ml at 5 ml/h was started intraoperatively.

**Fig. 2:**
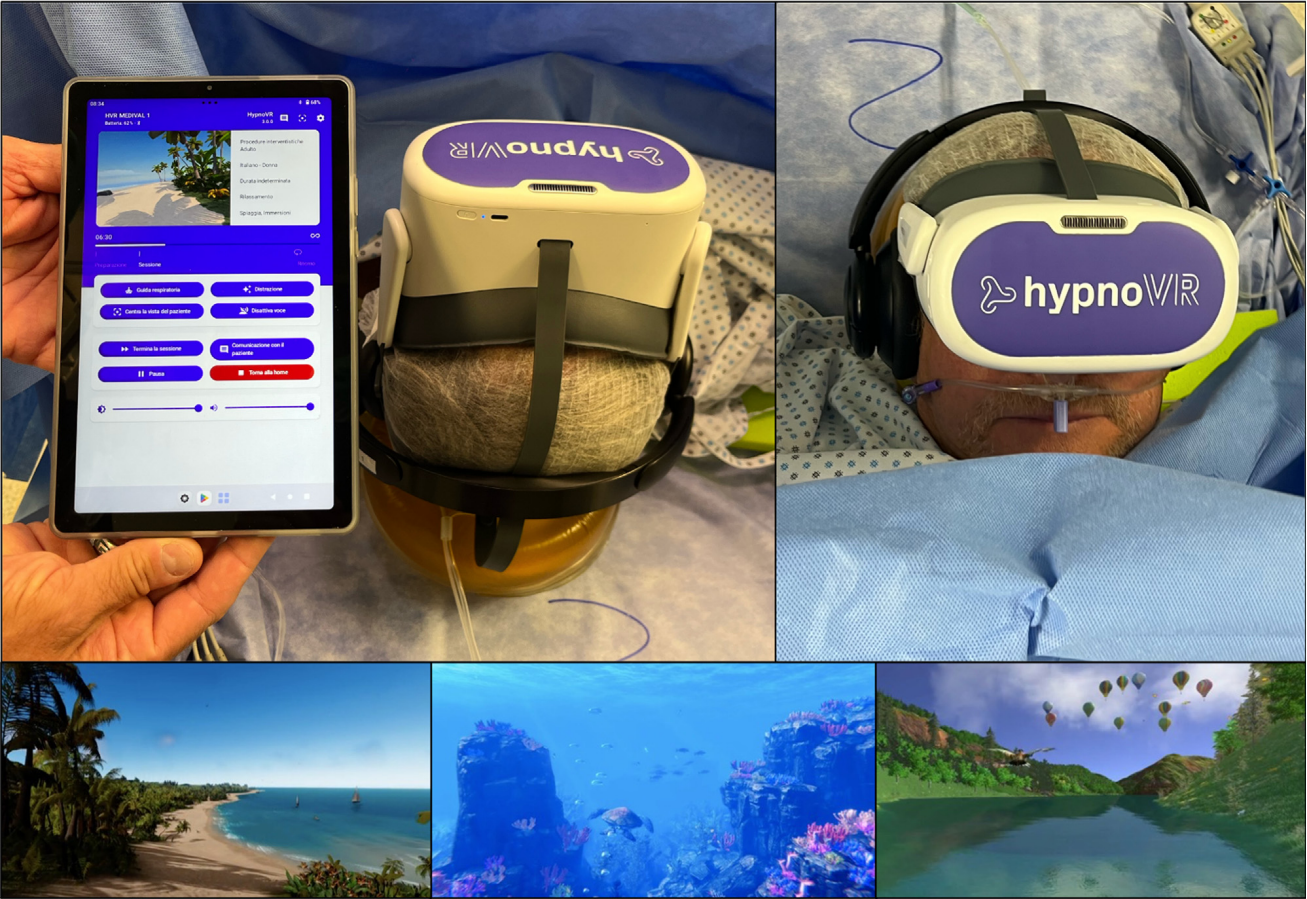
HypnoVR system consisting of three components: a headset, integrated headphones, and a tablet interface. The headset is shown mounted on the patient in the operating room, while the tablet displays the immersive virtual reality (VR) environment selected by the patient. The lower row shows three examples of VR scenarios available to the patient during the procedure.

The HypnoVR system features a head-mounted display combined with an integrated audio headset that provides an immersive audiovisual environment aimed at diverting the patient’s attention from the ongoing procedure. The system also includes a tablet via which the surgeon or anesthesiologist can interact with the patient during their navigation experience to gather information. Patients were asked to select their preferred immersive scenario before the surgical procedure (Fig. 2). The device was positioned and activated a few minutes before the start of the procedure, and was removed at the end, or at the patient’s request.

The patient was placed in a supine position with a 5° Trendelenburg tilt. A 3–5-cm incision was made 5 cm above the pubic symphysis, and the abdominal layers were dissected to expose and incise the anterior rectus fascia. The extraperitoneal space was developed via blunt dissection using an index finger and a balloon dissector. The SP access-port device was placed, and CO_2_ insufflation was maintained via an AirSeal system. An additional 5-mm assistant port was inserted 3–5 cm lateral to the main incision on the right side.

**Fig. 3:**
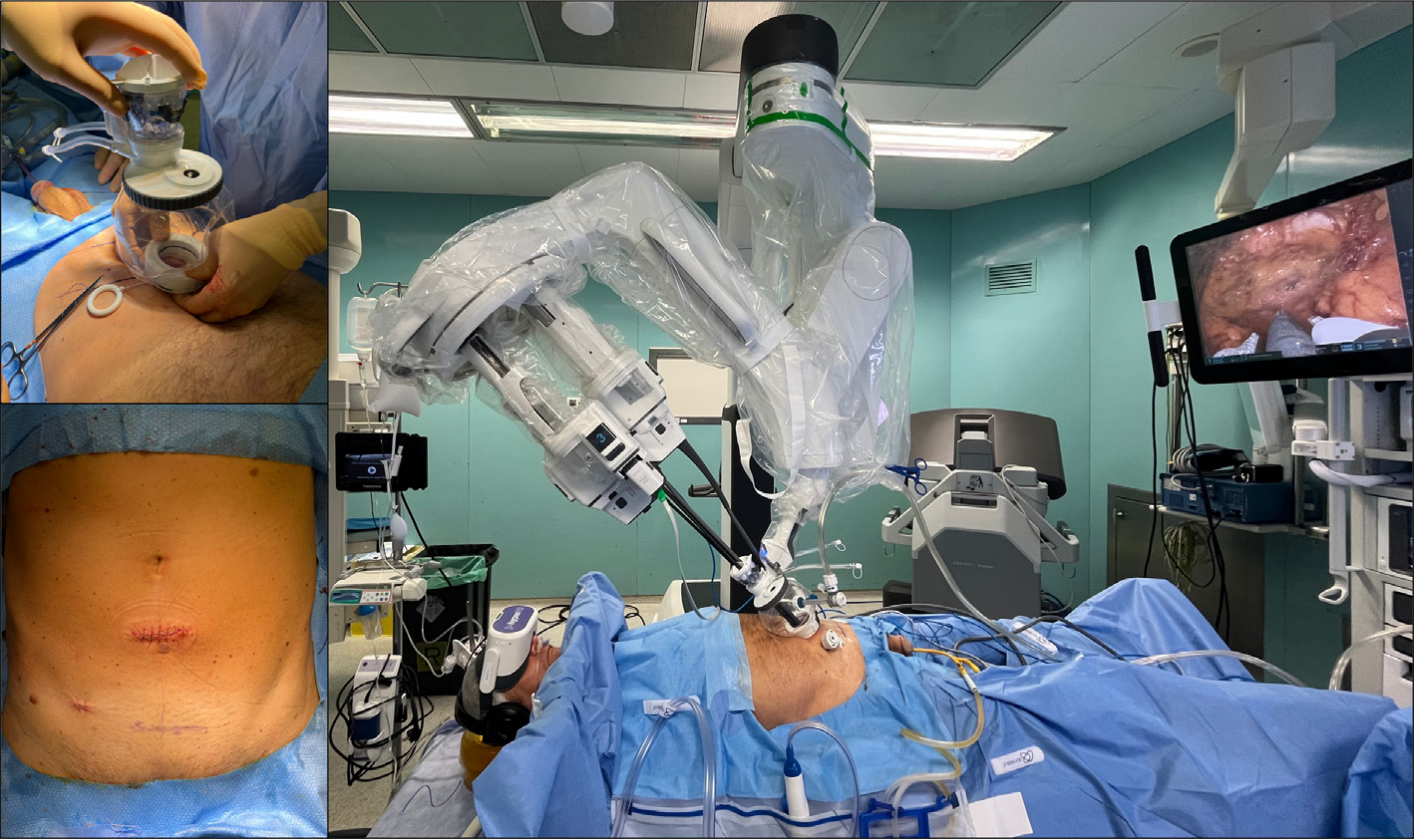
Intraoperative setting for VR-assisted neuraxial anesthesia during extraperitoneal single-port robot-assisted radical prostatectomy. Images show the patient awake under spinal anesthesia with the VR headset, the single-port device in place with the robot docked, and the final cosmetic outcome after suprapubic incision. VR = virtual reality.

For robotic instrumentation, the camera was placed at the 12-o’clock position, Maryland bipolar forceps at the 9-o’clock position (left hand), Cadiere forceps at the 6-o’clock position, and monopolar curved scissors at the 3-o’clock position (right hand). Dissection and reconstruction followed the same principles as for conventional multiport RARP, which ensured a familiar workflow in the extraperitoneal space (Fig. 3).

Data were collected for patient demographics, anesthesiological and surgical outcomes, and VR visor–related parameters. Demographics covered age, BMI, ASA status, and prior surgeries. Anesthesiological outcomes included anesthesia type, sedation, pain, and adverse events. Surgical outcomes included operative time, blood loss, hospital length of stay, and complications. Visor outcomes focused on tolerability, comfort, distraction, and subjective experience, measured using validated questionnaires.

Before admission and just before surgery, patients completed the State-Trait Anxiety Inventory (STAI), which assesses stable anxiety (trait, STAI-T) and situational anxiety (state, STAI-S) via 40 Likert-scale items [7].

Intraoperatively, pain was assessed via a 10-point Visual Analog Scale (VAS). Immediately after surgery, patients completed the STAI-S and VAS for anxiety. At 24 h after surgery, anxiety was reassessed using the STAI-S questionnaire, and the usability of the device was evaluated according to the Health Information Technology Usability Evaluation Scale (Health-ITUES) (Supplementary material) [8]. The first set of questions evaluated the impact of the headset on comfort, anxiety reduction, and ease of use. The second set focused on immersive realism, comfort, and potential use in other medical settings.

Ten patients were enrolled in this case series, with a median age of 66 yr (interquartile range [IQR] 60.5–70.2), median prostate volume of 32.5 ml (IQR 25–55.5), and median preoperative prostate-specific antigen of 9 ng/ml (IQR 6.5–11.5). All patients had localized low-risk prostate cancer, of International Society of Urological Pathology grade 1 in 30% and grade 2 in 70% of cases. A single surgeon experienced in multiport robotic surgery and more than 100 prior SP robotic prostate procedures performed all the procedures. Additional characteristics are summarized in Table 1.

**Table 1:** Patient characteristics

Parameter	Result
Median age, yr (IQR)	66.0 (60.5–70.2)
Median body mass index, kg/m^2^ (IQR)	24.0 (22.2–25.7)
Median ASA score (IQR)	2 (2–2.2)
Median Charlson Comorbidity Index (IQR)	4.5 (3–5)
Prior abdominal surgery, *n* (%)	
None	5 (50)
Appendectomy	3 (30)
Umbilical hernioplasty	1 (10)
Inguinal hernioplasty	1 (10)
Median prostate volume, ml (IQR)	32.5 (25–55.5)
Median preoperative PSA, ng/ml (IQR)	9 (6.5–11.5)
Prostate cancer clinical stage, *n* (%)	
cT1c	5 (50)
cT2a	3 (30)
cT2b	2 (20)
Gleason score at biopsy, *n* (%)	
6 (3 + 3)	3 (30)
7 (3 + 4)	7 (70)
Median maximum lesion size at MRI, mm, (IQR)	12 (8–15)
Extracapsular extension, *n* (%)	0 (0)

IQR = interquartile range; ASA = American Society of Anesthesiologists; PSA = prostate-specific antigen; MRI = magnetic resonance imaging.

Neuraxial anesthesia (CSE) was predominantly administered at the L1–L2 level (70%). No rescue additional doses of epidural ropivacaine were administered. There were no differences in the pCO_2_ level or respiratory rate between median baseline and maximal intraoperative values. Two episodes of mild hypotension requiring a vasoactive agent were recorded; no episodes of bradycardia occurred during any of the procedures.

The median pain score was 0/10 at discharge from both the operating room and the recovery room discharge. No patient exhibited movements during surgery, and no procedure needed any interruption (Supplementary material).

The median operative time was 90 min, and the median hospital stay was 2 d. No intraoperative or postoperative complications were recorded (Supplementary material).

Nine patients (90%) used the visor throughout the procedure, while one (10%) requested its removal because of intolerance. Another patient (10%) reported insufficient acoustic isolation, although he completed the procedure with the visor (Table 2).

**Table 2:** HypnoVR-related characteristics

Parameter	Result
SP-RARP completed with the patient wearing a HypnoVR headset, *n* (%)	9 (90)
Complete isolation from the external environment, *n* (%)	9 (90)
Median STAI-T score before admission (IQR)	41 (36–52.75)
Median STAI-S score (IQR)	
Before admission	40 (36.5–50.5)
Before surgery	51 (48–65.75)
Immediately after surgery	43.5 (35.75–47.75)
24 h after surgery	38.5 (34.25–44)
Median VAS score (IQR)	
Before surgery	61.5 (46.5–70.75)
During surgery	49.5 (38.25–52.5)
Immediately after surgery	50 (32.75–54.25)

SP-RARP = single-port robot-assisted radical prostatectomy; IQR = interquartile range; STAI-T = State-Trait Anxiety Inventory-Trait; STAI-S = State-Trait Anxiety Inventory-State; VAS = Visual Analog Scale.

**Fig. 4:**
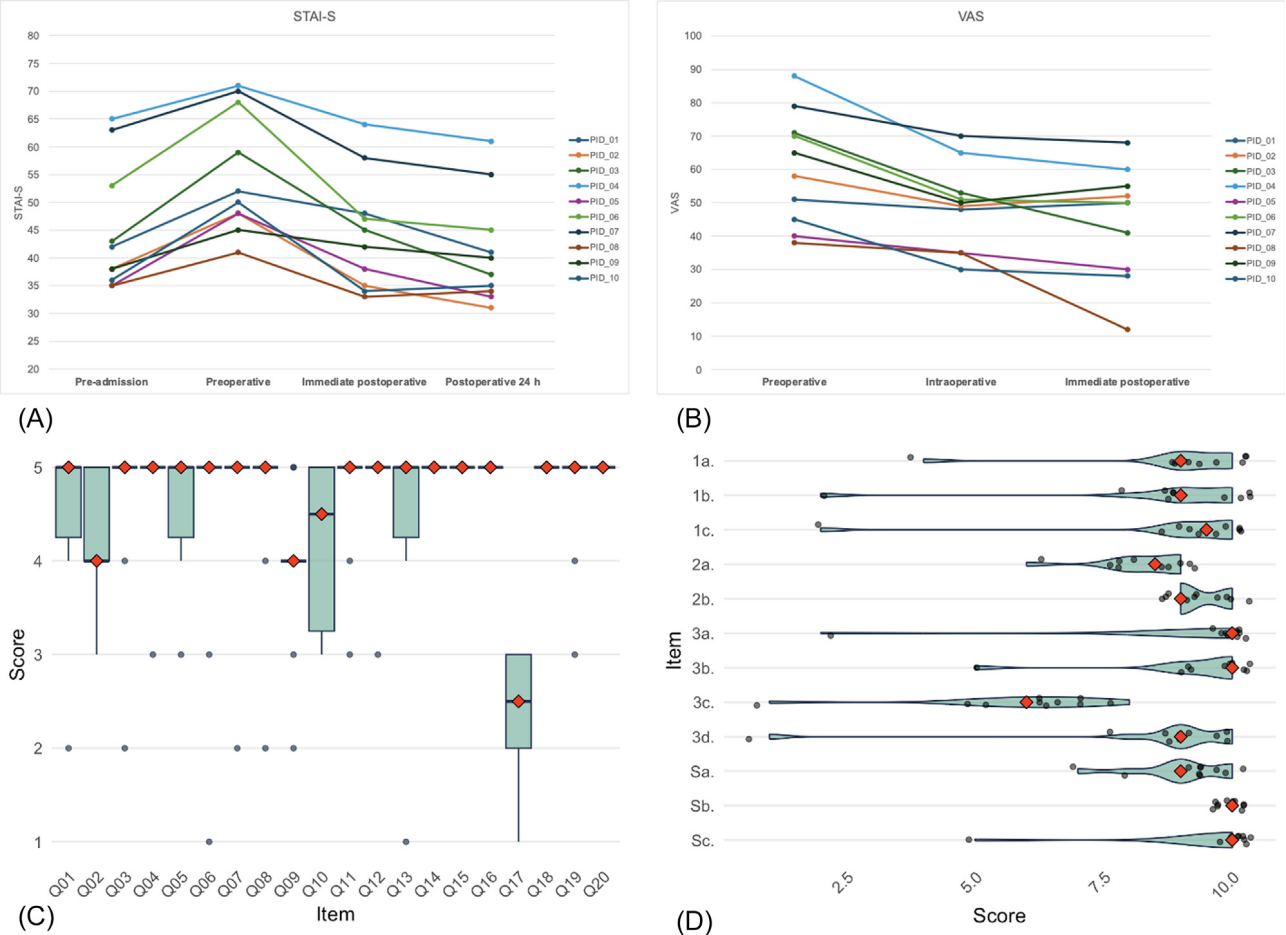
STAI-S, VAS, and Health-ITUES results. (A) Individual patient responses to (A) the STAI-S questionnaire and (B) the VAS questionnaire across time points. (C) Box plot representation of scores for the first 20 items of the Health-ITUES questionnaire. Blue boxes indicate the distribution of responses, while red diamonds represent median values. (D) Violin plot showing the distribution of responses to the Detailed Evaluation of VR Experience section of the Health-ITUES questionnaire. Red diamonds indicate median values. ITUES = Information Technology Usability Evaluation Scale; PID = patient identity; STAI-S = State-Trait Anxiety Inventory-Sate; VAS = Visual Analog Scale; VR = virtual reality.

Regarding patient perception, median STAI-S scores revealed significantly higher preoperative anxiety (51 points) in comparison to pre-admission (41 points). Anxiety decreased immediately after surgery (43.5 points) and even further by 24 h after surgery (38.5 points) to a lower level than at baseline (Fig. 4A).

VAS anxiety scores revealed similar trends, with a higher median preoperative anxiety score (61.5 points) in comparison to intraoperative (49.5 points) and postoperative (50 points) levels (Fig. 4B).

Health-ITUES questionnaire responses indicated general patient satisfaction with the visor, with a median score of 9/10, although comfort and wearability received lower scores, with one outlier patient requesting removal because of intolerance (Fig. 4C,D).

## 2 Discussion

Taken together, our results support the combination of neuraxial anesthesia and perioperative VR support for extraperitoneal SP-RARP to minimize the overall impact of surgery on patients. In our case series of patients with PC, the combination of these technologies was associated with excellent perioperative outcomes, including a median operative time of 90 min, negligible blood loss, and a short median hospital stay of 2 d. Importantly, neuraxial anesthesia provided effective analgesia with minimal hemodynamic fluctuations, no intraoperative patient movements, and no need for conversion to general anesthesia, confirming the feasibility of such an approach in minimally invasive urological surgery [9].

From a patient-centered perspective, VR support during SP-RARP was highly valued, as in other urological settings [10]. Most patients reported that the immersive environment reduced intraoperative anxiety, provided a distraction from the surgical setting, and enhanced their sense of control over the procedure. Quantitative assessment results supported these perceptions, showing a significant reduction in anxiety from preoperative levels to 24 h after surgery. In addition, responses to the Health-ITUES questionnaire indicated high overall acceptance and usability of the VR system, although a minority of patients noted minor discomfort with wearability and limited acoustic isolation.

Our study is not devoid of limitations, such as the small sample size and the single-center, single-surgeon design, which may limit generalizability and introduce selection or performance biases. Furthermore, the single-arm study lacked a control group, so there is a need for future controlled trials to confirm these preliminary findings. Moreover, patients with claustrophobia and those who refused to wear the VR headset were excluded, which may have introduced a selection bias toward less anxious individuals.

Nevertheless, this represents the first study demonstrating the feasibility and safety of such a technological combination for major surgery. The integration of neuraxial anesthesia and VR for extraperitoneal SP-RARP represents a technological, patient-centered framework to reduce surgical invasiveness and physiological stress during both the procedure itself and postoperative recovery.

  ***Conflicts of interest***: The authors have nothing to disclose.
