# Supplementary material for: Single-port Robotic Prostatectomy with Neuraxial Anesthesia and Virtual Reality Support: Combining Technologies To Minimize Surgical Impact
Source: Eur Urol Open Sci. 2025 Nov 24;83:30–5. doi: 10.1016/j.euros.2025.11.003 (PMC12686644; doi:10.1016/j.euros.2025.11.003)
Supplement: Supplementary Data 1 [file mmc1.docx]

### **Health Information Technology Usability Evaluation Scale (Health-ITUES)**

### Questionnaire: Patient Experience with the VR Headset During Surgery

This questionnaire aims to gather your opinion on your experience with the VR headset during the surgical procedure. You will find a series of statements. For each statement, indicate how much you agree by choosing a number from 1 to 5, where:

1 = Strongly disagree 2 = Disagree 3 = Neither agree nor disagree 4 = Agree 5 = Strongly agree

There are no right or wrong answers, only your personal impressions matter.

###

### IMPACT

1. I think the VR headset was a positive addition to my experience during the surgery.
2. I think the VR headset made the surgery more tolerable for me.
3. The VR headset helped reduce my anxiety and worry during the surgery.

###

### PERCEIVED UTILITY

1. Using the VR headset helped me relax during the surgery.
2. Using the VR headset made the duration of the surgery feel shorter.
3. Thanks to the VR headset, I focused less on the noises and environment of the operating room.
4. The VR headset was useful for improving my overall experience during the surgery.
5. The VR headset contributed to making my experience more enjoyable.
6. Thanks to the VR headset, I was able to “take my mind off things” and distract myself in a positive way.
7. The VR headset helped me isolate myself from the routine in a pleasant way.
8. Using the VR headset improved the quality of my experience (in terms of comfort and well-being).
9. The VR headset helped me manage my emotional experience during the surgery.

###

### EASE OF USE

1. I felt comfortable using the VR headset.
2. It was easy to understand how to use the VR headset.
3. It was simple to follow the steps indicated by the system.
4. It was easy to interact with external staff.
5. The VR headset was not uncomfortable to wear.

###

### USER CONTROL

1. When I received input from the staff, it was clear and easy to understand what I needed to do.
2. In case of a temporary interruption of the immersive experience, I was able to resume easily.
3. The information and instructions I received about using the VR headset were clear.

## Detailed Evaluation of VR Experience

For the following questions, indicate your answer using a number from 1 to 10, where:

1 = Not at all 10 = Absolutely yes

You can also use intermediate values (2–9) to indicate the degree of your opinion. Again, there are no right or wrong answers; the important thing is to express your experience honestly.

###

### Q1. Overall Usefulness

1a. How useful do you find the VR headset for improving your experience during surgery compared to standard medical and nursing support alone?

1b. Do you think the VR headset could be helpful in reducing the patient’s anxiety related to the surgery?

1c. Do you think the VR headset could be helpful in reducing the perceived duration of the surgery for the patient?

###

### Q2. Accuracy and Realism

2a. How realistic and accurate do you find the natural landscapes shown in the VR headset (sea, forest, mountains, sky)?

2b. How appropriate do you find the music and ambient sounds paired with the VR landscapes?

###

### Q3. Experience During Surgery

3a. How useful do you find the VR headset as a tool for distraction and relaxation during surgery?

3b. How much do you think the type of immersive environment chosen can influence the type of experience?

3c. How comfortable do you think the VR headset is for the patient to wear?

3d. How adequate do you think the conclusion of the immersive experience is in terms of a gradual return to reality?

### Qs. Specific Questions

Sa. Did using the VR headset give you the perception of being more supported by the surgical team compared to the standard procedure?

Sb. Did you find the time and dialogue with the staff adequate to choose your specific immersive experience?

Sc. How interesting do you find the possibility of using the VR headset for other moments (e.g., rehabilitation, postoperative recovery in the ward)?
