# Supplementary material for: Single-port Robotic Prostatectomy with Neuraxial Anesthesia and Virtual Reality Support: Combining Technologies To Minimize Surgical Impact
Source: Eur Urol Open Sci. 2025 Nov 24;83:30–5. doi: 10.1016/j.euros.2025.11.003 (PMC12686644; doi:10.1016/j.euros.2025.11.003)
Supplement: Supplementary Data 2 [file mmc2.docx]

Supplementary File 2: Perioperative anesthesiologic characteristics

| Neuraxial anesthesia  Epidural Space, num (%)   - L1-L2 - L2-L3   Subarachnoid Space, num (%)   - L1-L2 - L2-L3 | 8 (80%)  2 (20%)  7 (70%)  3 (30%) |
| --- | --- |
|  |  |
| Injection, num, median (IQR) | 1 (1;1) |
| Time of injection, min, median (IQR) | 9.23 (8.82;12.42) |
| Movements during surgery, num (%) | 0 (0) |
| Procedure interruption, num, num (%) | 0 (0) |
| RASS, median (IQR) | -1 (-3;-1) |
| Episode of hypotension, num (%) | 2 (20) |
| Episode of reversible hypotension, num (%) | 2 (20) |
| Episode of bradycardia, num (%) | 0 (0) |
| Episode of reversible bradycardia, num (%) | 0 (0) |
| Initial arterial pCO2, mmHg, median (IQR) | 39 (39;39) |
| Initial respiratory rate, num per minute, median (IQR) | 16 (12;16) |
| Maximal arterial pCO2 (mmHg), median (IQR) | 40.5 (38.2;42.7) |
| Maximal respiratory rate, num per minute, median (IQR) | 16 (14;18) |
| Intraop. IVFs given, mL, median (IQR) | 800 (700;800) |
| Intraop. levobupivacine dose, mg, median (IQR) | 5 (5;5) |
| Max intraop. dexmedetomidine dose (mcg/kg/h), median (IQR) | 0.6 (0.6;0.7) |
| Total intraop. fentanyl dose received, mcg, median (IQR) | 0 (0;0) |
| Intraop. propofol infusion, mg, median (IQR) | 0 (0;0) |
| Intraop. ropivacaine dose addition, mg, median (IQR) | 0 (0;0) |
| Bromage at OR discharge, Median (IQR) | 3 (3-3) |
| Pain score at OR discharge (NRS), median (IQR) | 0 (0;0) |
| Pain score at recovery room discharge (NRS), median (IQR) | 0 (0;0) |

IQR: interquartile range; RASS: Richmond Agitation-Sedation Scale; pCO2: Partial pressure of carbon dioxide; IVSs: intravenous fluids; OR: operatory room; NRS: Numeric Rating Scale
