# Supplementary material for: Single-port Robotic Prostatectomy with Neuraxial Anesthesia and Virtual Reality Support: Combining Technologies To Minimize Surgical Impact
Source: Eur Urol Open Sci. 2025 Nov 24;83:30–5. doi: 10.1016/j.euros.2025.11.003 (PMC12686644; doi:10.1016/j.euros.2025.11.003)
Supplement: Supplementary Data 3 [file mmc3.docx]

Supplementary File 3: Perioperative surgical data

| Operative time, min, Median (IQR) | 90 (80;100) |
| --- | --- |
| Type of Nerve-Sparing, num (%)   - Intrafascial - Interfascial - Extrafascial | 4 (40%)  5 (50%)  1 (10%) |
| Bladder-neck preservation, num (%)   - Yes - No | 3 (30%)  7 (70%) |
| Pneumo pressure, mmHg, Median (IQR) | 6 (6;6) |
| Estimated Blood Loss, mL, Median (IQR) | 600 (500;750) |
| Intraoperative complications, num (%) | 0 (0%) |
| Hospital stay, days, Median (IQR) | 2 (2;2) |
| First flatus, days, Median (IQR) | 1 (1;1) |
| Postoperative complications, num (%) | 0 (0) |
| Catheterization time, days, Median (IQR) | 6 (5;7) |
| Pathological stage, num (%)   - pT2 - pT3a - pT3b | 4 (40%)  4 (40%)  2 (20%) |
| Surgical Margin status, num (%)   - R0 - R1 | 8 (80%)  2 (20%) |
| Continence at 1 week, pads num, Median (IQR) | 1 (0;1.7) |
| Continence at 1 month, pads num, Median (IQR) | 0 (0;0) |
| Potency at 1 month, num (%)   - Yes - No | 3 (30%)  7 (70%) |
